# Jawbones Scaffold Constructed by TGF-β1 and BMP-2 Loaded Chitosan Microsphere Combining with Alg/HA/ICol for Osteogenic-Induced Differentiation

**DOI:** 10.3390/polym13183079

**Published:** 2021-09-13

**Authors:** Yongxin Tan, Liqun Zhang, Muhammad Shahid Riaz Rajoka, Zhanhua Mai, Ali Bahadur, Hafiza Mahreen Mehwish, Muhammad Umair, Liqing Zhao, Yiguang Wu, Xun Song

**Affiliations:** 1 College of Chemistry and Environmental Engineering, Shenzhen University, Shenzhen 518060, China; tanyonxin@126.com (Y.T.); shahidrajoka@yahoo.com (M.S.R.R.); joshua_mai@163.com (Z.M.); umair_uaf@hotmail.com (M.U.); 2 Department of Stomatology, Shenzhen Union Medical Hospital of Huazhong University of Science and Technology (Sixth Affiliated Hospital of Shenzhen University), Shenzhen 518060, China; zliqun23@163.com; 3 School of Pharmaceutical Science, Health Science Center, Shenzhen University, Shenzhen 518060, China; mahreen.mehwish@yahoo.com; 4 Food and Feed Immunology Group, Laboratory of Animal Food Function, Graduate School of Agricultural Science, Tohoku University, Sendai 980-8572, Japan; 5 Department of Transdisciplinary Studies, Graduate School of Convergence Science and Technology, Seoul National University, Seoul 16229, Korea; alibahadur138@gmail.com

**Keywords:** chitosan microsphere, osteogenic differentiation, jawbone’s scaffold, biocompatibility, osteogenic-induced differentiation

## Abstract

Bone scaffolds based on multi-components are the leading trend to address the multifaceted prerequisites to repair various bone defects. Chitosan is the most useable biopolymer, having excellent biological applications. Therefore, in the present study, the chitosan microsphere was prepared by the ion–gel method; transforming growth factor β (TGF-β1) and bone morphogenetic protein 2 (BMP-2) were loaded onto it and then combined with alginate/hyaluronic acid/collagen (Alg/HA/ICol) to construct a jawbones scaffold. The Alg/HA/ICol scaffolds were characterized by FTIR and SEM, and the water content, porosity, tensile properties, biocompatibility, and osteogenic-induced differentiation ability of the Alg/HA/ICol jawbones scaffolds were studied. The results indicate that a three-dimensional porous jawbone scaffold was successfully constructed having 100–250 μm of pore size and >90% of porosity without cytotoxicity against adipose-derived stem cells (ADSCs). Its ALP quantification, osteocalcin expression, and Von Kossamineralized nodule staining was higher than the control group. The jawbones scaffold constructed by TGF-β1 and BMP-2 loaded chitosan microsphere combining with Alg/HA/ICol has potential biomedical application in the future.

## 1. Introduction

The bone is a solid tissue consisting of organic materials (35%) and inorganic material (65%), which are accountable for their elasticity and strength [1]. Hence, numerous biomaterials such as silicate, bioactive glasses, hydroxyapatite, and chitosan-based materials have been industrialized to mimic the composition and functionality of bone [2]. Amongst them, the chitosan-based biopolymers have attracted interest due to their excellent bioactivities such as biocompatibility and bioavailability [3,4,5].

Bone tissue defects caused by maxillofacial tumor, trauma, and disease along with various other factors are a common problem in orthopedics [6], which are usually solved by autologous bone graft, allograft, and xenograft [7]. However, the source of autogenous bone is limited, and there is immune rejection of allogeneic and heterogeneous bones. So, these problems might be solved by bone tissue engineering technology with scaffold materials, signaling molecules (or growth factors), and seed cells [8,9].

A scaffold is the basic framework of bone tissue engineering with demanding requirements for its biocompatibility and mechanical properties [10,11]. Collagen, sodium hyaluronate, and sodium alginate have been proven to be good biomaterials for bone scaffolds [12,13,14]. Among them, Type I collagen is a kind of biomaterial derived from natural bone as the most abundant form of collagen in the human body, and it has a very low immunogenicity compared to other immunogenic proteins due to its stability and unique triple helix structure [15].

In addition to osteoinductivity, the constructed scaffold should have an extremely porous structure having interconnected pores along with appropriate mechanical stiffness, which leads to enhance cell-to-cell interaction and exchange of nutrition to promote the growth of bone [16]. So, the electrospinning might be considered as an effective technique to manufacture the interconnected scaffold having a highly porous structure and surface area [17,18]. The BMPs are potent osteoinductive factors that have the potential to induce the osteoblast differentiation and bone formation [19,20].

As an important component of chondrocytes’ extracellular matrix, sodium hyaluronate has excellent biocompatibility, higher structural porosity, and larger surface area among numerous biomaterials, and it plays a vital role in promoting the interaction between cells and other organisms, regulating cell differentiation and division, making it an ideal bone tissue engineering material [21]. Sodium alginate is a kind of natural polysaccharide from brown algae, which has excellent biomechanical properties, biocompatibility, and low immunogenicity. However, strong hydrophilicity makes it difficult to generate sufficient adhesion to cells and influence the biological behavior of cells [22]. The good adsorption to cells of collagen and sodium hyaluronate makes up for the deficiency of sodium alginate, which could increase the mechanical strength of a simple HA-COL composite scaffold. Therefore, Alg/HA/ICol is a suitable scaffold material for bone tissue engineering applications.

Studies [13] have shown that when BMP is combined with TGF-β, BMP enhances the osteogenic capacity of TGF-β, while TGF-β increases the induced capacity of BMP [23]. Natural chitin is chemically modified to obtain chitosan with good biocompatibility, good biodegradability, non-antigenicity, and non-toxic effects. It can be used to prepare drug-loaded microspheres for binding and protecting growth factors as well as slow-releasing drugs [24,25]. Adipose tissue-derived mesenchymal stem cells (ADSCs) have the potential to differentiate into osteocytes and chondrocytes under in vitro induced conditions.

Based on our previous studies on the adsorption of TGF-β1 and BMP-2 by the chitosan microsphere, the chitosan microsphere was prepared by the ion–gel method; TGF-β1 and BMP-2 were loaded on it and then combined with Alg/HA/ICol to construct a jawbones scaffold. The basic properties of Alg/HA/ICol and jawbones scaffolds were detected. ADSCs were inoculated on the jawbones scaffold to evaluate its biocompatibility and osteogenic induced differentiation ability. Therefore, the present study was useful to provide reference for the in vitro culture of tissue engineering bone and the selection of repairing materials for bone defect.

## 2. Materials and Methods

### 2.1. Preparation and Characterization of Alg/HA/ICol Scaffolds

#### 2.1.1. Preparation of Alg/HA/ICol Scaffolds

Alg (Macklin Biochemical Co., Ltd., Shanghai, China), HA (Macklin Biochemical Co., Ltd., China) and ICol (Macklin Biochemical Co., Ltd., Shanghai, China) were mixed in a mass ratio of 5:1:1. The 10.0 mL of Alg hydrosol was regulated to be 0.05%, 0.10%, 0.15%, 0.20%, and 0.25% (*w*/*w*), and pH 7.4, respectively. Then, 10 mL, 5 g/L of calcium chloride solution was added into the above hydrosols for cross-linking, respectively. The formative hydrogels were freeze-dried in a SCIENTZ-18N freeze-dryer (Ningbo Xinzhi Biochemical Co., Ltd., Ningbo, China) to obtain three-dimensional porous Alg/HA/ICol scaffolds.

#### 2.1.2. Characterization of Alg/HA/ICol Scaffolds

The IR spectra of the scaffold materials were obtained by an IR Affinity-1 Fourier Transform infrared spectrometer (Perkin, TE, USA). The surface morphology and aperture size of the scaffolds were observed by a S-3400N scanning electron microscope (Hitachi, Tokyo, Japan) after gold-spraying treatment with the acceleration voltage of 15 kV. The scaffolds were cut out to be samples with a length of 10 mm and a width of 10 mm, and their tensile properties were tested by a CMT4304 electronic tensile machine (MTS, Perkin, TX, USA) with the tensile rate of 10 mm/min.

#### 2.1.3. Swelling Ratio of Alg/HA/ICol Scaffolds

After drying the surface of a saturated water-absorbing scaffold with filter paper, it was weighed to be *W_t_*. Then, it was freeze-dried and weighed to be *W*_0_. The swelling ratio of scaffold was calculated according to Equation (1) [24].

(1) $$\mathrm{Swelling}\;\mathrm{ratio}\;(\%)\;=\;\frac{W_{t}-W_{0}}{W_{0}}\times 100\%$$

#### 2.1.4. Porosity of Alg/HA/ICol Scaffolds

A certain quality (*W*_1_) of freeze-dried scaffold was immersed into the volumetric flask containing ethanol with vacuum water pumping until no bubbles at the surface of scaffold were observed. The weight of the volumetric flask containing ethanol and freeze-dried scaffold was *W*_2_. Taking out the scaffold, the weight of the volumetric flask containing the residual ethanol was *W*_3_, and the porosity of the scaffold was calculated according to Equation (2) [26].

(2) $$\mathrm{Porosity}\;(\%)=\frac{W_{2}-W_{3}-W_{1}}{W_{2}-W_{3}}\times 100$$

### 2.2. Preparation and Characterization of TGF-β1 and BMP-2 Loaded Chitosan Microsphere Combining with Alg/HA/ICol Jawbones Scaffold

First, 0.5 g of chitosan (Jiangxi Golden brilliance Medical Products Co., Ltd., Yichun, China) was dissolved in 500 mL, 1% (*w*/*w*) of acetic acid solution to obtain a 1.0 mg/mL of chitosan solution. Taking 200 mL of above chitosan solution in a beaker, its pH value was adjusted to be 5.0 with dilute NaOH solution; then, 40 mL, 1 mg/mL of sodium tripolyphosphate solution was slowly dripped into the chitosan solution under continuous magnetic stirring. After the dripping was completed, the suspension was obtained by magnetic stirring for 1 h at room temperature. Taking 20 mL of the above suspension, 4.0 mL, 10 ng/mL of BMP-2 (ProSpec, Jerusalem, Israel) solution and 2.5 mL, 100 mg/mL of TGF-β1 (ProSpec, Israel) solution were added into with continuous magnetic stirring at 4 °C respectively. Then, 7 h later, the mixed suspension was completely shifted to 10 mL of Alg/HA/ICol hydrosol with 0.1% (*w*/*w*) of Alg, which was then cross-linked by adding 10 mL, 5.0 g/L of calcium chloride solution for 15 min. The prepared hydrogel was freeze-dried in a Scientz-18N freeze-dryer to obtain the three-dimensional porous jawbones scaffold, whose structure and performance were characterized as same as in Section 2.1.

### 2.3. Testing of Cytotoxicity and Osteogenic Induction

Adipose-derived stem cells (ADSCs) of well-grown mice (Cyagen, Santa Clara, CA, USA) were taken and digested into single-cell suspension by adding an appropriate amount of pancreatic enzyme (GENVIEW, Hollister St Ste 520, Houston, TX, USA), and the cell suspension was diluted by using medium. The number of cells inoculated in each well of 96-well plates was approximately 1 × 10^5^/well. The cells were cultured in a 5% of CO_2_ incubator at 37 °C for 24 h until adherence. The experimental cells were divided into the control group and the scaffold-treated group. After adherence at 24 h, the Alg/HA/ICol scaffold was added into scaffold group cells and cultured in an incubator. Cells were taken for CCK-8 detection at 2-, 4-, 8-, 12-, and 16-day intervals. The cells were rinsed with PBS twice and then added with 10% (*v*/*v*) of CCK-8 solution (Dojindo, Kumamoto, Japan) in the medium for further culture for 4 h. The absorbance (OD450) was determined by using an Epoch enzyme-labeled instrument (BioTek, Winuschi, VT, USA).

For osteogenic induction, the cells were cultured according to the above methods, the jawbones scaffold was added to the scaffold group cells for culture in a 5% CO_2_ incubator at 37 °C for 24 h; then, the original medium was replaced with osteogenesis induction medium every 3 d once. At 0, 5-, 7-, 10- and 14-days’ culture, the cells were rinsed with PBS twice, fixed with 4% (*w*/*w*) of paraformaldehyde for 30 min, and then washed with PBS once again for the coming osteogenesis induction experiments, including the observation of cell morphology by the inverted microscope (MOTIC, Berlin, Germany). The cells were stained by hematoxylin–eosin staining (HE, Nanjing Jiancheng Biotechnology Co., Ltd., Nanjing, China), sealed with neutral balsam (Sinopharm Chemical Reagent Co., Ltd., Shanghai, China), and then, the growth of the cells was observed by an inverted microscope and photographed. The alkaline phosphatase (ALP) detection kit (Nanjing Jiancheng Biological Technology Co., Ltd., Nanjing, China) was used to detect the ALP cell activity. The liquid supernatant in medium was collected after centrifugation at 14,000 RPM for 15 min and added with standard fluid and chromogenic reagents. After bath and coloring, it was detected for OD520 using an enzyme-labeled instrument (BioTek, Winuschi, VT, USA); then, the data were converted into the King unit. The Alizarin Red staining liquor (Servicebio, Wuhan, China) was used for staining for 5 min; then, the mineralized nodules were washed by running water and observed under an inverted microscope. The cytotoxicity of the jawbones scaffold was measured by the CCK-8 method. The 10% (*v/v*) of CCK-8 solution in the culture medium was added to each well and continued in the cell incubator for 4 h; then, its OD520 was measured by an enzyme-labeled instrument (BioTek, Winuschi, VT, USA). The expression of osteocalcin mRNA was detected by RT-PCR. The total RNA was extracted by the Trizol method, and its concentration and purity were determined by an ultra-micro ultraviolet analyzer (Quawell Technology, San Jose, CA, USA). The total RNA was converted to cDNA by a reverse transcription kit (DBI, Berlin, Germany), and fluorescence quantitative PCR amplification was performed. Primer sequences are shown in Table 1, and the relative gene expression was calculated with 2-ΔΔCt.

### 2.4. Statistical Analysis

SPSS 22.0 software was used for data processing, and ANOVA, a Welch amendment test, and a K-W test were used for comparison tests. *p* < 0.05 was considered as significant difference, while *p* > 0.05 was considered as no significant difference.

## 3. Results

### 3.1. Structure and Performance of Alg/HA/ICol Scaffolds

**Figure 1:**
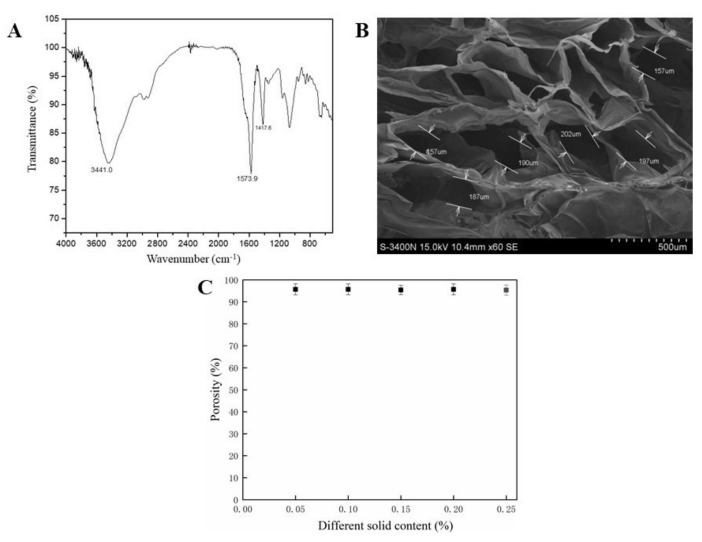
FTIR spectrum (**A**), SEM image (**B**), and Porosity (**C**) of Alg/HA/ICol scaffold.

The FTIR spectrum of the Alg/HA/ICol scaffold prepared from the hydrosol with 0.1% (*w*/*w*) of Alg is shown in Figure 1A. The strong absorption peak at 3441 cm^−1^ in the figure belonged to the characteristic absorption peak of hydroxyl groups in the range of 3700–3000 cm^−1^, reflecting the large amount of hydroxyl groups in Alg and HA. The spectrum peak at 1610 cm^−1^ corresponded to the amide Ⅰ band of ICol, and the spectrum peak at 1400 cm^−1^ corresponded to its amide Ⅱ band. The amide Ⅰ band showed obvious displacement, indicating the molecular interaction between ICol and other molecules, which was indicated by some studies [27,28,29] to be that between ICol and HA.

The SEM image of the cross-section of the Alg/HA/ICol scaffold prepared from the hydrosol with 0.1% (*w*/*w*) of Alg is shown in Figure 1B. It could be seen that the internal aperture of the scaffold was interlinked, mainly circular, and elliptic, and its diameter was within the range of 100~250 μm. Studies showed that the appropriate scaffold pore size range for growing bone cells was 75–250 μm, while the recommended pore size range was 200–300 μm for fibrocartilage tissue culture. Furthermore, the connected pore (>100 μm) structure provided nutrients exchange with the surrounding environment, supplies, and metabolites excretion, which was conducive to the formation of tissue structure [30,31]. Therefore, the aperture size and connected structure of the Alg/HA/ICol scaffold are suitable for bone cell culture.

The porosities of Alg/HA/ICol scaffolds prepared from the hydrosols with different Alg contents are shown in Figure 1C. The porosities of all the freeze-dried scaffolds were uniform, which were higher than 90%, meeting the requirement of tissue engineering a scaffold with a porosity of higher than 80%.

The swelling ratios of Alg/HA/ICol scaffolds prepared from the hydrosols with different Alg contents are shown in Table 2. With the increase in Alg content, the swelling ratio of the scaffold presented a decreasing trend, indicating that the hydrophilic swelling performance of the scaffold decreased.

The tensile properties of Alg/HA/ICol scaffolds prepared from the hydrosols with different Alg contents are shown in Table 3. It could be seen that the tensile strength of the scaffold increased with the increase in Alg content, while the elongation at break of the scaffold decreased slightly, reflecting the contribution of Alg to the mechanical strength of the Alg/HA/ICol scaffold.

### 3.2. Cytotoxicity of Alg/HA/Icol Scaffold

**Figure 2:**
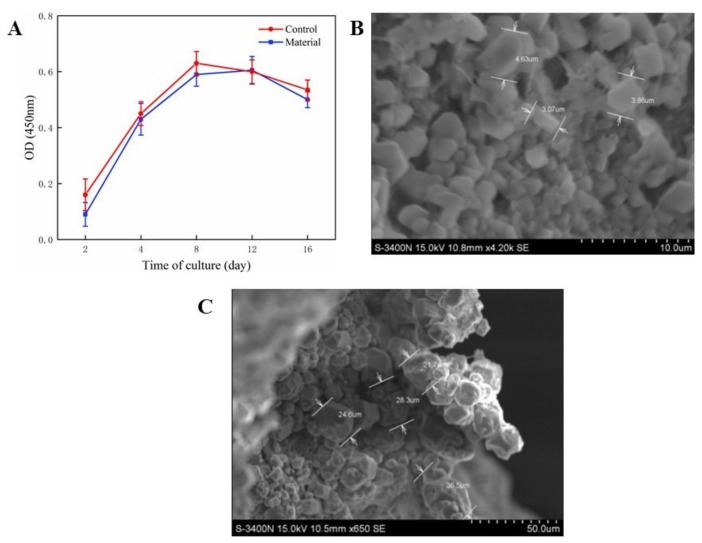
Cell proliferation curves (**A**), SEM images of ADSCs cultured on the scaffold surface for 4 d (**B**) and 8 d (**C**).

The cell proliferation curves were obtained by the CCK-8 method, as shown in Figure 2A. The cell proliferation curve of the Alg/HA/ICol scaffold prepared from the hydrosol with 0.1% (*w*/*w*) of Alg was not significantly different from that of the control, indicating that the scaffold had good biocompatibility and the combination of the three biomaterials had no adverse effect on the proliferation of ADSCs.

SEM images of ADSCs cultured on the scaffold surface for 4 d and 8 d are shown in Figure 2B, respectively. After 8 days’ culture, the cells on the scaffold surface showed a certain growth trend, and their diameter increased five to eight times compared with that on the 4th day.

### 3.3. Structure and Performance of Jawbones Scaffold

**Figure 3:**
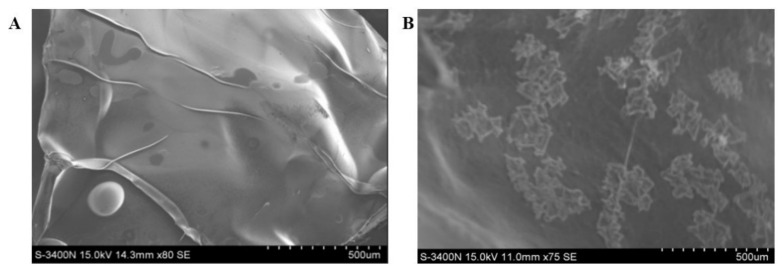
SEM images of bare scaffolds (**A**) and scaffolds combined with chitosan microspheres (**B**).

Figure 3 shows the differences in both scaffold surfaces under SEM. It can be seen that the scaffold without composite microspheres was smoother. After compounding the chitosan microspheres, the polymer microspheres were dispersed on the original smooth surface.

Table 4 compared the structure and mechanical properties of the Alg/HA/ICol scaffold prepared from the hydrosol with 0.1% (*w*/*w*) of Alg and the jawbones scaffold prepared by adding TGF-β1 and BMP-2-loaded chitosan microsphere into the Alg/HA/ICol scaffold. It could be seen that the hydrophilic swelling property of the jawbones scaffold decreased due to the addition of solid chitosan microspheres, so the swelling ratio of the jawbones scaffold decreased. Chitosan particles were dispersed in the scaffold framework, so the porosity of the jawbones scaffold was slightly lower than that of the Alg/HA/ICol scaffold. Furthermore, the research results showed that the addition of chitosan particles had little effect on the mechanical properties of the Alg/HA/ICol scaffold.

### 3.4. Cytotoxicity and Osteogenic Induction of Jawbones Scaffold

**Figure 4:**
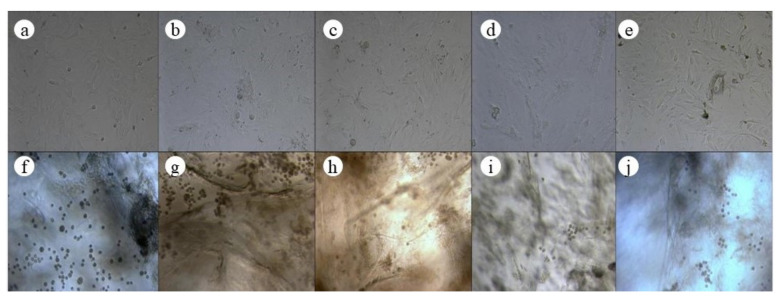
Microscopic images of ADSCs at different times (**a**–**e**: the jawbones scaffold group; **f**–**j**: the control group) (×20).

The microscopic photos of ADSCs at different times are shown in Figure 4, in which a–e are the microscopic photos of the cells in the jawbones scaffold group at 0 d, 5 d, 7 d, 10 d, and 14 d, and f–j were the microscopic photos of the cells in the control group at 0 d, 5 d, 7 d, 10 d, and 14 d. It could be seen that the morphology of the cells in the jawbones scaffold group was similar to that of the control group at different times of bone induction.

**Figure 5:**
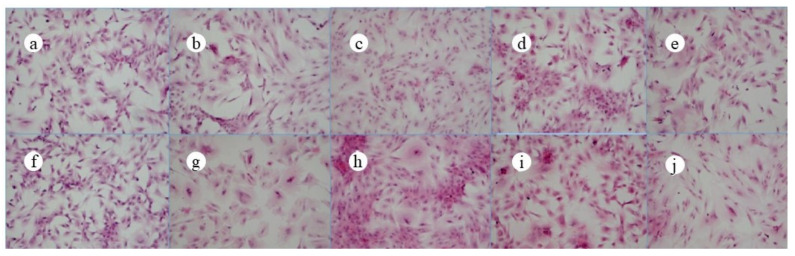
HE staining photos of ADSCs at different times (**a**–**e**: the jawbones scaffold group; **f**–**j**: the control group) (×40).

Figure 5 shows the HE staining photos of ADSCs at different times, in which a–e are the HE staining photos of the cells in the jawbones scaffold group at 0 d, 5 d, 7 d, 10 d, and 14 d, and f–j are the HE staining photos of the cells in the control group at 0 d, 5 d, 7 d, 10 d, and 14 d. As shown in Figure 3, the morphology of the cells in the jawbones scaffold group was roughly the same as that of the control group at different times of bone induction.

**Figure 6:**
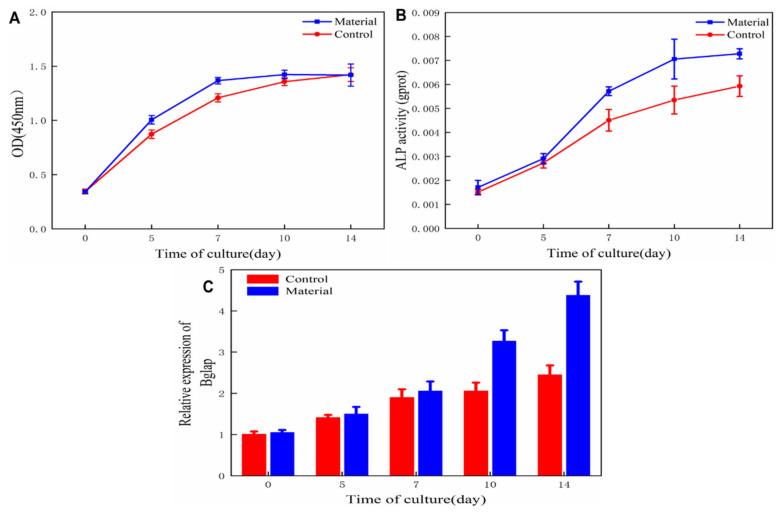
Cell proliferation rate (**A**), cell ALP activity (**B**), and relative mRNA expression level of cell osteocalcin (**C**) of the scaffold treated group and control group.

The cell proliferation curves obtained by the CCK-8 test are shown in Figure 6A. The early proliferation rate of ADSCs on the jawbones scaffold was higher than that on the control, which proved that the scaffold was important for cell culture and was conducive to cell adhesion, proliferation, and nutrition transmission. Since the culture medium and cell inoculation density were the same in both groups, the cell proliferation rates of the two groups tended to be the same after 10 days’ cell proliferation. Most importantly, the jawbones scaffold showed no cytotoxicity at all.

The quantitative detection of cell ALP can reflect the differentiation level of pre-osteoblasts [32] activities of bone-induced cells on the jawbones scaffold and the control were detected by ELISA, as shown in Figure 6B. After 5 days’ bone induction, the ALP activity of cells cultured on the jawbones scaffold was significantly higher than that of cells on the control group, proving that bone-induced factors in the jawbones scaffold played an effective role in enhancing the cell ALP activity and significantly promoting the differentiation of pre-osteoblasts into mature osteoblasts.

Cell osteocalcin expression level is the most commonly used indicator of terminal osteoblast differentiation [31]; the relative mRNA expression levels of cell osteocalcin with different times of bone induction on the jawbones scaffold and the control are shown in Figure 5c. The relative mRNA expression levels of osteocalcin of two groups of cells increased gradually with bone-induced culture time. After 7 d, the relative mRNA expression level of osteocalcin of cells on the jawbones scaffold increased remarkably and showed an apparent difference from that on the control. It showed that a large number of late-mature osteoblasts emerged on the later jawbones scaffold toward terminal differentiation.

**Figure 7:**
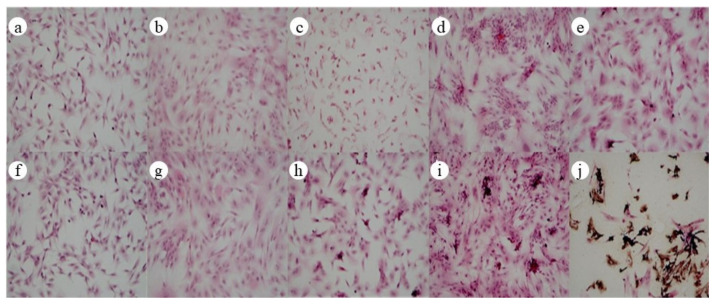
Staining images of cellular Von Kossa mineralized nodules in bone induction at different times (**a**–**e**: the control group; **f**–**j**: the jawbones scaffold group) (×40).

Figure 7 shows the staining images of cellular Von Kossa mineralized nodules in bone induction at different times, in which a–e are the images of the control group at 0 d, 5 d, 7 d, 10 d, and 14 d, and f–j are the images of the jawbones scaffold group at 0 d, 5 d, 7 d, 10 d, and 14 d. A small number of black sedimentary nodules were observed on the jawbones scaffold 10 d after bone induction; its color deepened and the amount increased significantly in 14 d after bone induction. It implied that a large amount of calcium-like matrix had been produced and mineralized, and the jawbones scaffold significantly stimulated the formation of cellular mineralized nodules.

## 4. Conclusions

A TGF-β1 and BMP-2-loaded chitosan microsphere was compounded with Alg/HA/Icol to construct a jawbones scaffold. The research results showed that the jawbones scaffold could form a suitable three-dimensional porous space and could directionally induce the osteogenic differentiation of ADSCs. The present results suggested that the constructed jawbone scaffold possessed strong bone repair capability and might have potential application in bone tissue engineering. The in vitro results suggested that the proposed jawbone scaffold had low cytotoxicity and it was beneficial to the adhesion and proliferation of adipose-derived stem cells (ADSCs) cells. Furthermore, the proposed jawbone scaffolds having bone repaired functions might be consider as promising candidates for jaw bone repair. This study was prospective to be applied in clinically repairing jawbones’ defect and solving the patients’ pain. However, still, further study is needed to explore the potential clinical application of jawbone in bone regeneration.

## Figures and Tables

**Table 1:** Primer parameters of qPCR.

ID	Sequence (5′-3′)	Product Length (bp)
β-actin F	CATTGCTGACAGGATGCAGA	139
β-actin R	CTGCTGGAAGGTGGACAGTGA	
Bglap F	TTCTGCTCACTCTGCTGACC	203
Bglap R	AGGGTTAAGCTCACACTGCT	

**Table 2:** Swelling ratios of Alg/HA/ICol scaffolds.

Alg Content in Hydrosol (wt %)	Swelling Ratio (%)
0.05	75.46 ± 0.3
0.10	72.56 ± 0.3
0.15	72.23 ± 0.2
0.20	45.60 ± 0.2
0.25	41.59 ± 0.2

**Table 3:** Tensile properties of Alg/HA/ICol scaffolds prepared from the hydrosols with different Alg contents.

Alg Content in Hydrosol (wt %)	Tensile Strength (MPa)	Elongation at Break (%)
0.05	0.028 ± 0.001	29.7 ± 0.1
0.10	0.036 ± 0.002	28.3 ± 0.1
0.15	0.044 ± 0.000	26.6 ± 0.1
0.20	0.052 ± 0.001	26.0 ± 0.1

**Table 4:** Comparison of the structure and mechanical properties of Alg/HA/ICol and the jawbones scaffolds.

Type	Swelling Ratio (%)	Porosity (%)	Tensile Strength (MPa)	Elongation at Break (%)
Alg/HA/ICol	72.56 ± 0.31	95.63 ± 0.86	0.036 ± 0.02	28.3 ± 0.1
jawbones	52.89 ± 0.25	90.62 ± 0.59	0.038 ± 0.02	28.9 ± 0.1

## Data Availability

Data will be provided on demand.
